# Bone marrow-derived macrophages distinct from tissue-resident macrophages play a pivotal role in Concanavalin A-induced murine liver injury via CCR9 axis

**DOI:** 10.1038/srep35146

**Published:** 2016-10-11

**Authors:** Takeru Amiya, Nobuhiro Nakamoto, Po-sung Chu, Toshiaki Teratani, Hideaki Nakajima, Yumi Fukuchi, Nobuhito Taniki, Akihiro Yamaguchi, Shunsuke Shiba, Rei Miyake, Tadashi Katayama, Hirotoshi Ebinuma, Takanori Kanai

**Affiliations:** 1 Division of Gastroenterology and Hepatology, Department of Internal Medicine, Keio University School of Medicine, Tokyo, Japan; 2 Research Unit/Frontier Therapeutic Sciences, Sohyaku, Innovative Research Division, Mitsubishi Tanabe Pharma Corporation, Yokohama, Japan; 3 Department of Stem Cell and ImmuneRegulation, Yokohama City University Graduate School of Medicine, Yokohama, Japan; 4 Department of Pathophysiology, Faculty of Pharmaceutical Sciences, Hoshi University, Tokyo, Japan

## Abstract

The fundamental mechanism how heterogeneous hepatic macrophage (Mφ) subsets fulfill diverse functions in health and disease has not been elucidated. We recently reported that CCR9^+^ inflammatory Mφs play a critical role in the course of acute liver injury. To clarify the origin and differentiation of CCR9^+^Mφs, we used a unique partial bone marrow (BM) chimera model with liver shielding for maintaining hepatic resident Mφs. First, irradiated mice developed less liver injury with less Mφs accumulation by Concanavalin A (Con A) regardless of liver shielding. In mice receiving further BM transplantation, CD11b^low^F4/80^high^ hepatic-resident Mφs were not replaced by transplanted donors under steady state, while under inflammatory state by Con A, CCR9^+^Mφs were firmly replaced by donors, indicating that CCR9^+^Mφs originate from BM, but not from hepatic-resident cells. Regarding the mechanism of differentiation and proliferation, EdU^+^CCR9^+^Mφs with a proliferative potential were detected specifically in the inflamed liver, and *in vitro* study revealed that BM-derived CD11b^+^ cells co-cultured with hepatic stellate cells (HSCs) or stimulated with retinoic acids could acquire CCR9 with antigen-presenting ability. Collectively, our study demonstrates that inflammatory Mφs originate from BM and became locally differentiated and proliferated by interaction with HSCs via CCR9 axis during acute liver injury.

The liver is a specific organ with continuous exposure to many pathogens and commensal bacterial products from the intestinal tract. Hence, strict regulation of foreign antigens and subsequent inflammation is essential for maintenance of hepatic homeostasis, resulting in immunological tolerance in the liver. A number of immune cell subsets, such as T lymphocytes, dendritic cells (DCs), and macrophages (Mφs), are critically involved in diverse hepatic immunological characteristics^1^,^2^. Above all, Mφs, which comprise approximately 20% of hepatic immune cells, play a key role during the initiation of hepatic inflammation.

Until recently, a central dogma for development of Mφs has been described based on the mononuclear phagocyte system concept, proposing that tissue-resident Mφs are terminally differentiated and rely on constant recruitment of bone marrow (BM)-derived blood monocytes^3^. However, recent fate-mapping studies revealed that, although they are organ-dependent, tissue-resident Mφs are primitively fate-determined cells from the yolk sac and can be clearly distinguished from *Myb*-dependent hematopoietic stem cells that reside in the fetal liver or BM^3^,^4^,^5^,^6^,^7^,^8^,^9^,^10^. These two distinct types of Mφs, tissue resident Mφs and hematopoietic stem cell derived recruiting Mφs, are functionally unique and non-complementary to each other^11^,^12^,^13^, and have thus been considered to have different immunological roles^14^,^15^. However, it has also been reported that there is crosstalk involving populational and functional overlaps between resident Mφs and recruited Mφs. For example, intestinal and dermal tissue-resident Mφs in mice are replenished by blood circulating monocytes^16^,^17^. Moreover, the tissue-resident and recruited Mφ subsets in the peritoneal cavity^18^, heart^19^, and other tissues coexist under both steady state and inflammation, and each subset can proliferate in parallel^10^,^11^,^20^. In the liver, it has been reported that both hepatic-resident Mφs (i.e. Kupffer cells) and BM-derived Mφs (i.e. hepatic resident BM-derived Mφs) reside in liver sinusoids under steady state^4^. In the case of *Listeria monocytogenes* infection causing necrosis of Kupffer cells or clodronate-induced artificial depletion, BM-derived monocytes contribute to repopulation of the tissue-resident Mφ population^21^,^22^,^23^. In addition, after acetaminophen-induced liver injury, BM-derived monocytes do not contribute to the tissue-resident Mφ pool, while Kupffer cells can proliferate in addition to recruited monocytes^13^. Regarding the functional aspect, recruited Mφs certainly serve as the main cell subset producing proinflammatory cytokines, while Kupffer cells also produce these cytokines at an earlier time point than recruited Mφs in general^24^,^25^,^26^. The discrepancy among these reports is considered to arise through differences in physiological conditions and organ specificity along with the heterogeneity of Mφs. However, these results suggest that Mφs are regulated to develop from either resident or recruited cells and complement each other, depending on the involvement of specific conditions, such as inflammation, infection, and regeneration.

Tissue-resident DCs have an analogous transcriptional pattern regardless of the tissue involved^27^, while tissue-resident Mφs share only a few unique gene expressions and the majority of their transcription patterns are particular to individual organs^28^. Although this diversity of transcriptional patterns is influenced by environmental signals, such as local cytokines and metabolites^7^, their roles in the regulation of Mφ differentiation have only just begun to be elucidated.

Concanavalin A (Con A)-induced hepatitis is a murine model of natural killer T and T cell-mediated acute hepatic injury. In this model, tumor necrosis factor (TNF)-α-producing inflammatory Mφs promote Th1 responses, leading to massive necrosis in the liver. Recently, we reported that C-C motif chemokine receptor (CCR) 9-expressing Mφs (CCR9^+^Mφs) play an important role in this model as well as in a murine fibrosis model^29^,^30^, and further found that the CCL25-CCR9 axis is critical for the pathogenesis of acute liver damage as well as other previously reported chemokine receptors, CCR1, CCR2, and CCR8^31^,^32^,^33^.

Generally, inflammatory Mφs have been believed to originate from the BM, based on demonstrations that BM transplantation (BMT) following total body irradiation (TBI) can replace the Mφs population in the BM but not in hepatic resident Mφs population that is resistant to radiation. However, this well-established belief might not represent the original steady situation, because TBI itself could cause a substantial hepatic inflammation and diminish the function of resident Mφs in terms of differentiation and proliferation^6^. Based on these backgrounds, we aimed to clarify the origin of CCR9^+^Mφs during acute liver injury using a unique murine liver-shielded radiation model to overcome the limitations described above. In addition, we report a novel mechanism for regulating the migration and proliferation of hepatic inflammatory Mφs via CCR9 axis from circulating monocytes during acute liver injury.

## Results

### CCR9-expressing Mφs do not pre-exist under steady state, but accumulate in the injured liver

**Figure 1:**
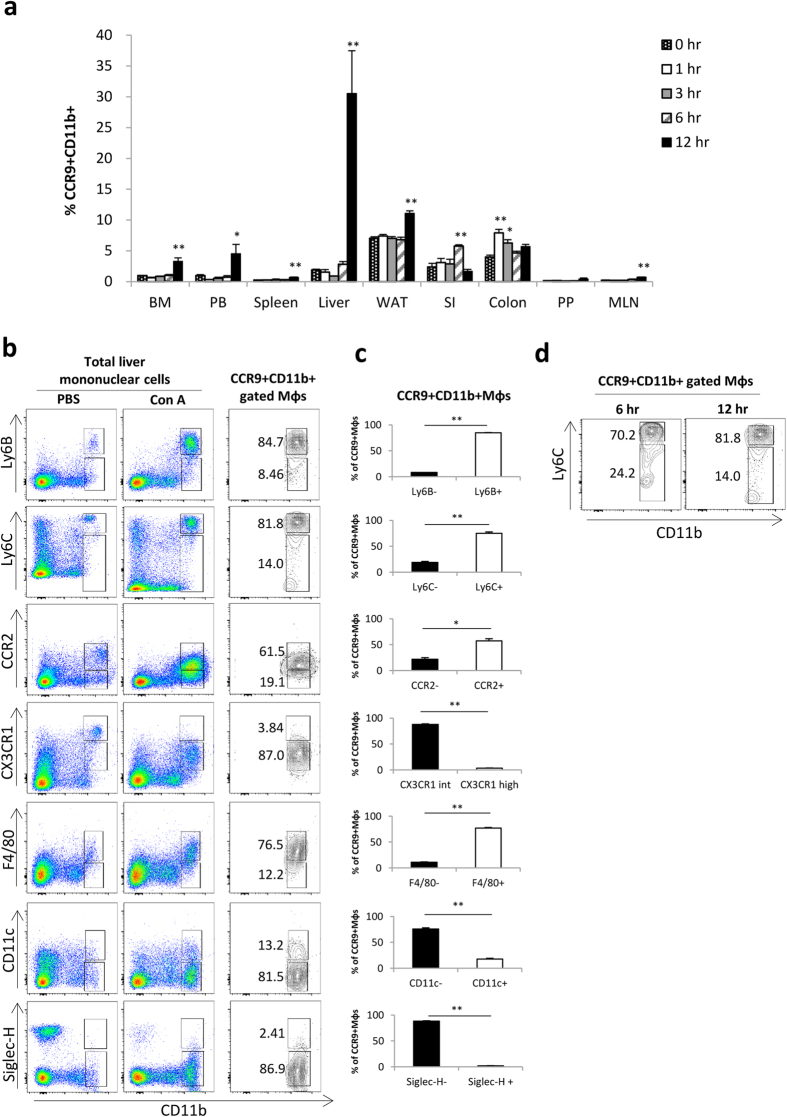
CCR9^+^Mφs are not pre-existing in steady state, but specifically accumulate to the injured liver. Mice were intravenously injected with Con A (20 mg/kg), and CCR9 expression in each immune cell subset was sequentially analyzed by flow cytometry. (**a**) Sequential change in the frequency of CCR9^+^CD11b^+^ Mφs in each organ. Data show mean ± SEM (n = 3). BM; bone marrow, PB; peripheral blood, WAT; white epididymal adipose tissue, SI; small intestine, PP; Peyer’s patch, MLN; mesenteric lymph node. (**b**) Phenotipic characterization of CD11b^+^ cells in PBS-injected total liver mononuclear cells (left), Con A-injected total liver mononuclear cells (middle), and ConA-injected hepatic CCR9^+^CD11b^+^ gated cells (right). (**c**) Percentage of each surface marker negative (left) and positive (right) cells in Con A-injeted hepatic CCR9^+^CD11b^+^ gated cells. (**d**) Representative staining of CD11b and Ly6C on hepatic CCR9^+^CD11b^+^ gated cells at 6 hours (left) and 12 hours (right) following Con A injection. Data show mean ± SEM (n = 4). *p < 0.05, **p < 0.01.

First, we investigated the sequential changes in the distribution of CCR9^+^Mφs in various tissues following Con A injection to clarify the possibility that CCR9^+^Mφs pre-exist in other tissues and migrate into the liver. CCR9^+^CD11b^+^Mφs appeared in the liver as early as 6 hours after Con A injection (Fig. 1 and Supplementary Fig. S1), consistent with our previous report^29^. Meanwhile, there were no dramatic increases in the frequency of CCR9^+^CD11b^+^Mφs in other tissues (Fig. 1 and Supplementary Fig. S1). Furthermore, pre-existing CCR9^+^CD11b^+^Mφs were not detected in any tissues under steady state. These results indicate that accumulation of CCR9^+^Mφs is specifically induced in the liver, and that pre-existing CCR9^+^Mφs are unlikely to migrate or proliferate in the inflamed liver following Con A injection. Further phenotypic analysis of CCR9^+^CD11b^+^Mφs emerged in the inflamed liver revealed that these cells are Ly6B^+^, Ly6C^+^, CCR2^+^, F4/80^+^, CX3CR1^int^, CD11c^−^, and Siglec H^−^, indicating that these cells are phenotypically monocytes-derived macrophages, not DCs (Fig. 1b–d).

### BM-derived macrophages are indispensable during the course of Con A-induced acute liver injury

**Figure 2:**
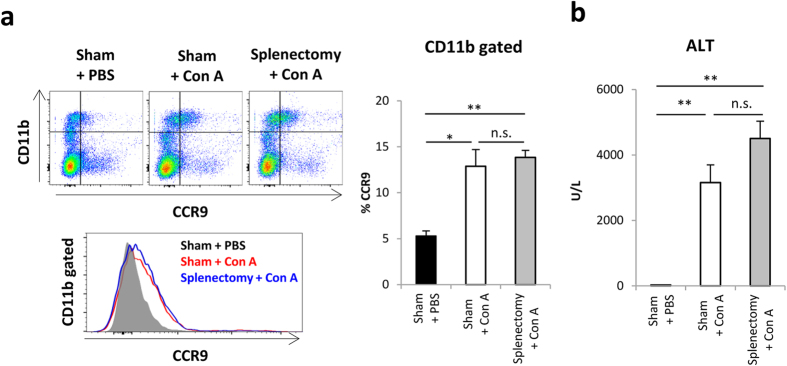
Splenic reservoir monocytes are not essential to induce hepatic injury. Mice were intravenously injected with Con A on Day14 after splenectomy or sham operation. (**a**) Left: Representative CD11b and CCR9 staining on total isolated mononuclear cells (upper), and CCR9 histogram on CD11b^+^ gated cells (lower) from Sham + PBS, Sham + Con A, and Splenectomy + Con A treated mice. Right: Percentage of CCR9^+^ cells in CD11b^+^ cells. Data show mean ± SEM (n = 3–5). (**b**) Serum ALT levels of mice in indicated groups. Data show mean ± SEM (n = 3–5). *p < 0.05, **p < 0.01. n.s.: not significant.

To investigate the possibility that splenic monocytes are the origin of CCR9^+^Mφs in the liver^34^, mice were treated with splenectomy or sham operation at 2 weeks before Con A injection. As shown in Fig. 2a,b, the frequency of CCR9^+^Mφs and the subsequent liver injury were not affected by splenectomy, suggesting that CCR9^+^Mφs were not derived from splenic monocytes.

**Figure 3:**
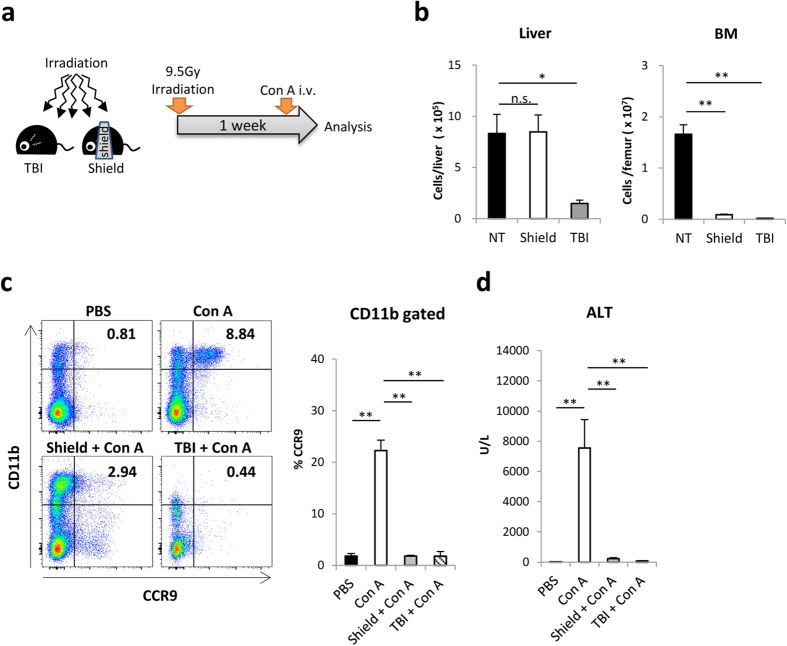
BM-derived macrophages are indispensable during the course of Con A-induced acute liver injury. Mice were irradiated to total body (TBI) or irradiated with lead shield over a liver (shield), and further injected with Con A on Day7. (**a**) Methodological scheme to establish TBI and liver-shielded mice. (**b**) Absolute numbers of hepatic mononuclear cells (left) and femur-derived BM cells (right) before Con A injection. Data show mean ± SEM (n = 3–5). (**c**) Left: Representative CD11b and CCR9 staining on isolated hepatic mononuclear cells of mice in indicted group 12 hours after Con A injection. Right: Percentage of CCR9^+^ cells in CD11b^+^ cells. (**d**) Serum ALT levels of mice in indicated groups. Data show mean ± SEM (n = 4). *p < 0.05, **p < 0.01. n.s.: not significant.

Next, we examined the contribution of hepatic-resident Mφs in promoting Con A-induced liver injury with CCR9^+^Mφs accumulation. To this end, we established a unique partial radiation model that enables the maintenance of a large amount of hepatic immune cells including CD11b^low^F4/80^high^ Kupffer cells and CX3CR1^+^CD11b^high^F4/80^low^ hepatic-resident perivascular cells^4^,^8^,^35^. For this, mice were treated with a lethal dose (9.5 Gy) of irradiation with a shielding lead plate that covered the whole liver (Fig. 3a). Both hepatic mononuclear cells and BM cells were dramatically diminished in the TBI-treated mice, while hepatic mononuclear cells, but not BM cells, were maintained to the same extent as those in non-irradiated mice in the liver-shielded mice (Fig. 3b). Of interest, the accumulation of CCR9^+^Mφs in the liver and the subsequent liver injury induced by Con A were significantly milder in both the liver-shielded mice and TBI-treated mice compared with non-irradiated mice (Fig. 3c,d), suggesting that BM-derived macrophages are indispensable regardless of the existence of tissue resident macrophages.

### CCR9^+^Mφs are derived from the BM via blood circulating monocytes

**Figure 4:**
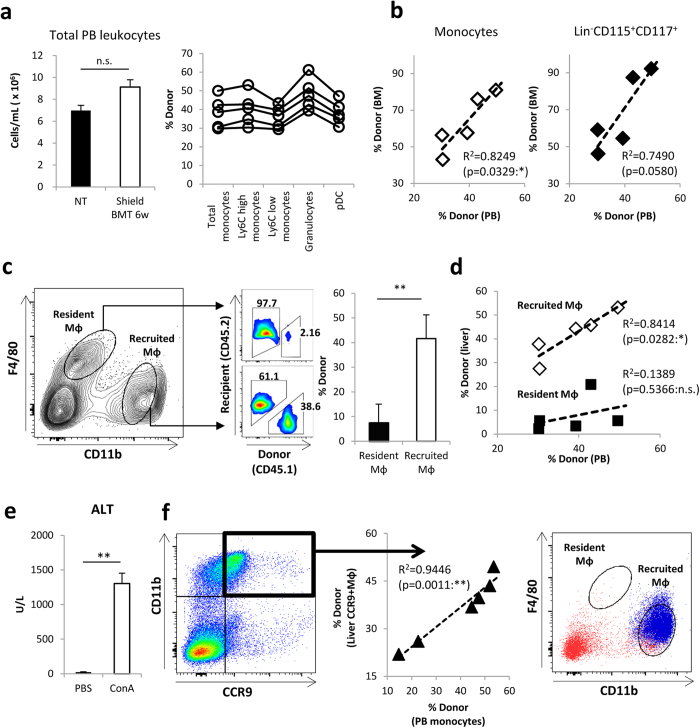
CCR9^+^Mφs are originated from BM via blood circulating monocytes without contribution of hepatic resident Mφs. Irradiated mice with a whole liver shield (shield) were reconstituted with BM cells. 6 weeks after BM transplantation, mice were further injected with Con A. (**a**) Left: Absolute cell numbers of total leukocytes in 1 ml PB of mice on 6 weeks after BMT. Right: The percentage of donor cells (chimerisms) in each cell fraction of PB. total monocytes: CD45^+^CD11b^+^Ly6G^−^Ly6C^++^, Ly6C high or low monocytes: CD45^+^CD11b^+^Ly6G^−^Ly6C^high or low^, granulocytes: CD45^+^CD11b^+^Ly6G^+^, plasmacytoid DC (pDC): CD45^+^CD11b^−^CD11c^+^. (**b**) Left: Correlation of the chimerism in monocytes fraction of PB and BM. Right: Correlation of the chimerism in monocytes fraction of PB and linage^-^CD115^+^CD117^+^ MDP-like cells fraction of BM. (**c**) Left: Representative staining of CD45.1 (donor cells) and CD45.2 (recipient cells) on CD11b^low^F4/80^high^ resident Mφs and CD11b^high^F4/80^low^ recruited Mφs in the liver of shielded BMT mice under steady state. Right: The percentage of donor-derived cells in resident Mφs and recruited Mφs. Data show mean ± SEM (n = 5). (**d**) Correlation between the chimerism of PB monocytes and the chimerism of recruited Mφs (white squares) or resident Mφs (black squares) in shielded BMT mice liver under steady state (n = 5 each) (**e**) Serum ALT levels of shielded BMT mice at 12 hours after Con A injection (inflammatory state). Data show mean ± SEM (n = 6). (**f**) Left: Correlation between the chimerism of PB monocytes and the chimerism of hepatic CD11b^+^CCR9^+^Mφs of shielded BMT mice under inflammatory state. Right: Representative CD11b and F4/80 staining on hepatic CD11b^+^CCR9^+^Mφs (blue dots) and whole hepatic cells (red dots). *p < 0.05, **p < 0.01. n.s.: not significant.

Based on the results shown in Fig. 3, we speculated that CCR9^+^Mφs were derived from outside the liver, BM. We verified this issue directly using partial BM chimeric mice, created by shielding the whole liver before irradiation and reconstituting with CD45-mismatched BM to distinguish hepatic-resident Mφs from BM-derived recruited Mφs as previous reported in peritoneum and pleura^11^,^18^. First, we traced the numerical dynamics of blood leukocytes after BMT to confirm BM reconstruction. The number of peripheral blood (PB) leukocytes was dramatically decreased at 2 days after BMT, began to be restored on day 7, and returned to the original level by 6 weeks (Fig. 4a left and Supplementary Fig. S2a). At this time point, the chimerism in various subsets of peripheral myeloid cells was confirmed (Fig. 4a right). There was a clear correlation between the chimerism of PB monocytes and that of CD11b^+^Ly6C^+^ monocytes or Lin^−^CD115^+^CD117^+^ immature macrophage dendritic cell progenitor (MDP)-like precursors in the BM (Fig. 4b), demonstrating that the BM was successfully reconstructed. In the liver of partial chimeric mice, the CD11b^high^F4/80^low^ fraction and CD11b^low^F4/80^high^ fraction were clearly distinguished (recruited Mφs and tissue-resident Mφs, respectively) at 6 weeks following BMT (steady state) (Fig. 4c left), consistent with previous reports^35^,^36^. Importantly, CD11b^low^F4/80^high^ tissue-resident Mφs were maintained with the recipient origin, while CD11b^high^F4/80^low^ recruited Mφs were chimeric (Fig. 4c right), and the chimerism was closely correlated with that in PB monocytes (Fig. 4d). These tissue-resident and recruited Mφs expressed cell markers of Kupffer cells (CD68, CD169)^35^ and inflammatory cells (Ly6B, CCR2)^3^,^37^, respectively (Supplementary Fig. S2b). Taken together, these results suggest that the partial chimeric model can enable us to discriminate hepatic-resident Mφs from recruited Mφs in the liver.

BM reconstituted mice at 6 weeks were further injected with Con A to elucidate the origin of CCR9^+^Mφs in the inflamed liver. As shown in Fig. 4e, partial chimeric mice developed acute liver injury by Con A, and CD11b^+^CCR9^+^Mφs emerged in the liver (Fig. 4f left). The chimerism of CCR9^+^Mφs was closely correlated with the individual chimerism in PB monocytes (Fig. 4f middle), and the majority of these populations were back-gated to the CD11b^high^F4/80^low^ fraction (Fig. 4f right). These results clearly indicate that CCR9^+^Mφs were derived from BM monocytes via blood circulating monocytes with little involvement of tissue-resident Mφs in the liver. Of note, even at an earlier time point when BM reconstitution has not been fully achieved, the chimerism of CCR9^+^ Mφs, but not CCR9^−^ Mφs was closely correlated with the individual chimerism in PB monocytes following Con A administration (Supplementary Fig. S2c).

We further confirmed the above results using clodronate-injected mice, in which resident macrophages are depleted. As shown in Supplementary Fig. S2d, the majority of CCR9^+^Mφs were detected in the liver following Con A administration in clodronate pre-injected mice, although statistically just a little fewer than uninjected mice.

### Recruited monocytes acquire CCR9 expression with local proliferation in the injured liver

**Figure 5:**
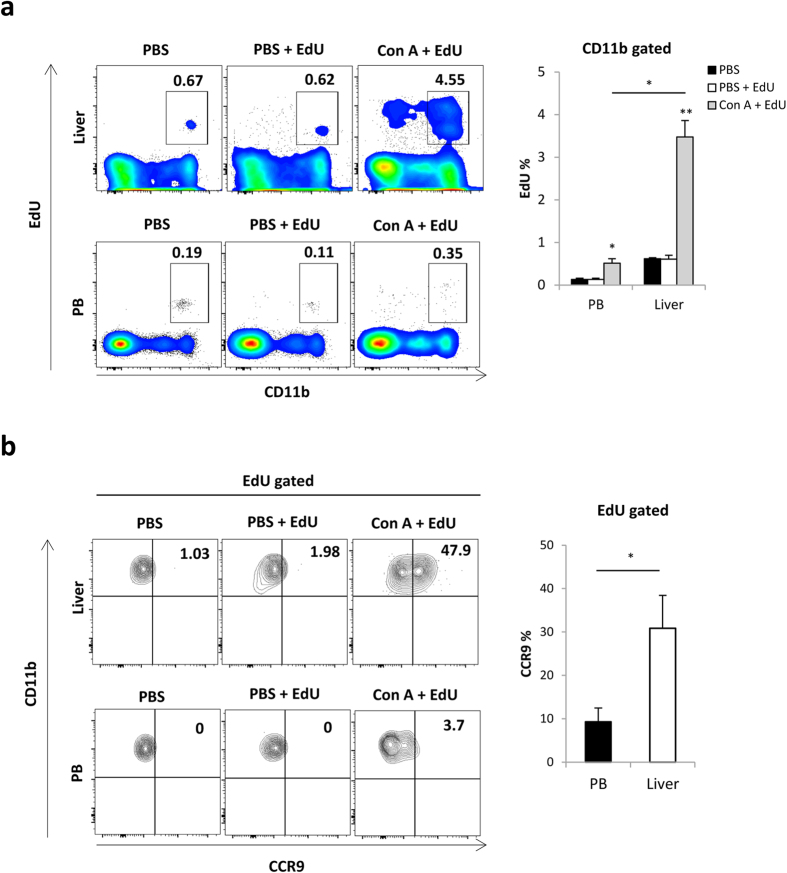
Recruited monocytes acquired CCR9 expression with local proliferation in the inflamed liver. PBS or Con A injected mice were intraperitonealy administered with EdU solution (50 mg/kg) 2 hours before sacrifice. (**a**) Left: Representative CD11b and EdU staining on whole mononuclear cells in the liver (upper) and PB (lower) from PBS, PBS + EdU, or Con A + EdU treated mice. Right: Percentage of EdU^+^ cells in CD11b^+^ gated PB or liver mononuclear cells. Data show mean ± SEM (n = 3–4). (**b**) Left: Representative CCR9 and CD11b staining on EdU^+^ gated cells in the liver (upper) and PB (lower) Right: The percentage of CCR9^+^ cells in EdU^+^ gated cells in PB and the liver of mice in indicated groups. Data show mean ± SEM (n = 3–4). *p < 0.05, **p < 0.01.

Next, we examined the mechanisms underlying how CCR9^+^Mφs proliferate during the process of Con A-induced acute liver injury. We quantitatively evaluated the proliferation of CD11b^+^Mφs in Con A-treated mice after intraperitoneal injection of EdU at 2 hours prior to dissection. As shown in Fig. 5a, a larger number of CD11b^+^Mφs in the liver of Con A-treated mice were EdU-positive compared with hepatic CD11b^+^Mφs in PBS-treated mice. Importantly, a smaller number of peripheral CD11b^+^ monocytes were proliferative, regardless of Con A injection. These results suggest that EdU^+^Mφs observed in the liver are not recently recruited from blood circulating monocytes, but proliferate in the inflamed liver. Importantly, almost half of the EdU^+^ (proliferative) Mφs expressed CCR9 in the liver, but not in PB (Fig. 5b). These results indicate, at least in part, that recruited monocytes acquired CCR9 expression with local proliferation in the inflamed liver.

### Upregulation of CCR9 expression in BM-derived monocytes, but not in hepatic mononuclear cells, is mediated by interaction with activated HSCs

**Figure 6:**
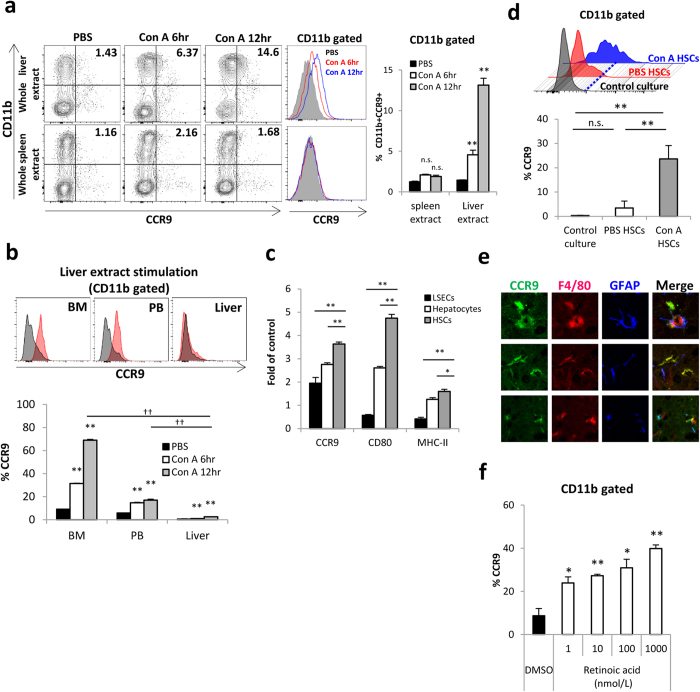
Differentiation into CCR9+Mφs in BM derived monocytes was mediated by interaction with activated HSCs. Total BM, PB, or liver mononuclear cells were cultured for 6 hours with extracts of whole liver or spleen from WT mice treated with PBS or Con A (6 hr or 12 hr). All extracts were prepared at same protein concentration (7 mg/mL). (**a**) Left: Representative CCR9 and CD11b staining on total BM cells cultured with whole liver extracts (upper) or spleen extracts (lower). Middle: Representative CCR9 expression on CD11b^+^ gated BM cells. Right: Percentage of CCR9^+^ cells in CD11b^+^ cells cultured with whole liver extracts or spleen extracts. Data show mean ± SEM of triplicate samples. (**b**) Upper: Representative histogram of CCR9 expression on gated BM, PB, or liver CD11b^+^ cells cultured with PBS or Con A injected liver extracts. Lower: Percentage of CCR9^+^ cells in BM, PB, or liver CD11b^+^ cells cultured with whole liver extracts. Data show mean ± SEM of triplicate samples. (**c**) Fold induction of CCR9, CD80, and MHC class II expression on CD11b^+^ gates BM cells cultured with extracts of LSECs, HSCs or hepatocytes from Con A-treated mice compared to those from PBS-treated mice. Data show mean ± SEM of triplicate samples. (**d**) Upper: Histogram of CCR9 expression on CD11b^+^ BM cells co-cultured with HSCs isolated from Con A or PBS treated mice for 4 days. Lower: Percentage of CCR9^+^ cells in BM CD11b^+^ cells co-cultured with HSCs. Data show mean ± SEM of triplicated samples. (**e**) Fluorescence immunohistochemistry of the liver from Con A treated mice. CCR9 (green), F4/80 (red) and GFAP (blue) were shown in a single immunofluorescence for each expression as well as merged co-immunofluorescence (yellow). (**f**) Percentage of CCR9^+^ cells in CD11b^+^ BM cells cultured with retinoic acid *in vitro* for 4 days. Data show mean ± SEM of triplicated samples. *p < 0.05, **p < 0.01, ^††^p < 0.01, n.s.: not significant.

To clarify the mechanism underlying how recruited BM-derived Mφs acquire CCR9 expression in the liver, total BM cells were cultured *in vitro* with whole liver or spleen extracts isolated from Con A-treated mice. CCR9 expression was enhanced in BM cells cultured with whole liver extracts isolated from Con A-administered mice, compared with BM cells cultured with liver extracts without Con A administration or spleen extracts regardless of Con A administration (Fig. 6a). Importantly, total BM and PB-derived CD11b^+^ cells, but not hepatic CD11b^+^ cells, had the capacity to express CCR9 (Fig. 6b). Collectively, these results indicate that BM-derived blood circulating monocytes differentiate into CCR9^+^Mφs in the surrounding area of the inflamed liver. Next, we investigated the specific cell subsets in the liver that mediate CCR9 upregulation in BM-derived monocytes. To this end, whole liver components from Con A-treated or PBS-treated mice were fractionated into LSECs, HSCs, and hepatocytes, and extracts of each fraction were cultured with BM-derived monocytes. BM-derived CD11b^+^ cells cultured with Con A-treated HSC extracts showed increased CCR9 expression along with class II and CD80 upregulation compared with CD11b^+^ cells cultured with extracts from LSECs or hepatocytes (Fig. 6c), indicating that BM-derived monocytes differentiate into pro-inflammatory Mφs with CCR9 acquisition by interaction with HSCs. Finally, total BM cells were co-cultured with HSCs to examine the direct interaction with activated HSCs. As shown in Fig. 6d, CD11b^+^ BM cells co-cultured with Con A-treated HSCs, but not PBS-treated HSCs, upregulated CCR9 expression. We also confirmed *in vivo* that CCR9 and F4/80 double-positive CCR9^+^Mφs were closely localized with GFAP^+^ HSCs, while CCR9-negative F4/80^+^Mφs were not (Fig. 6e). Furthermore, we confirmed that retinoic acids had the potential to increase CCR9 expression in BM cells as a contributing humoral factor of HSCs (Fig. 6f). These results indicate that activated HSCs with retinoic acids are the key cell subset that induce CCR9 expression in BM-derived monocytes in the liver.

## Discussion

**Figure 7:**
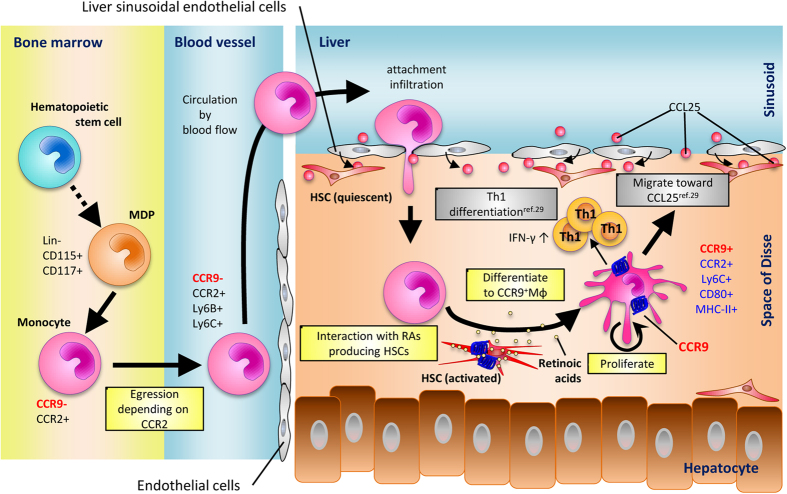
Proposed mechanism of origin and differentiation of CCR9^+^Mφs in Con A induced acute liver inflammation. Following Con A administration, CCR9 negative monocytes derived from MDP-like progenitors egress from BM depending on CCR2. Then, blood circulating monocytes from BM infiltrate into the injured liver by chemokines/cytokines/attachment dependent migration. Once monocytes infiltrate into the injured liver through sinusoidal lumen, monocytes interact with activated HSCs (e.g. retinoic acid from activated HSCs) and differentiate into CCR9^+^Mφs with a potential of proliferation as well as antigen presentation to T cells. Thus, CCR9^+^Mφs are originated from BM independently on whole heterogeneous hepatic resident Mφs (including Kupffer cells and also resident perivascular Mφs), and differentiated in injured liver with local proliferation to promote acute liver inflammation.

Inflammatory Mφs play a critical role in the initiation and development of liver injury, and in the subsequent liver fibrosis and carcinogenesis. We previously showed that CCR9^+^ inflammatory Mφs initiate acute liver injury through interaction with Th1 cells in the inflamed liver^29^, but the origin and precise mechanisms of the migration and proliferation of CCR9^+^Mφs has not been elucidated. The present study suggests a novel role for the CCR9 axis in the process of migration and differentiation of Mφs in the liver during acute liver injury, as summarized in Fig. 7.

Hepatic Mφs consist of hepatic-resident Mφs, widely known as Kupffer cells, and circulating/recruited Mφs. Previous reports showed that hepatic-resident Mφs activated by damage associated molecular patterns (DAMPs) or pathogen associated molecular patterns (PAMPs) secrete pro-inflammatory cytokines and recruit additional immune cells in the early phase of liver injury^15^,^38^. We initially asked whether hepatic-resident cells contribute to the initiation and development of acute liver injury in this murine model. For this purpose, we established a unique lead-shielding model that enables to protect the liver from irradiation. As expected, TBI-treated mice, in which both hepatic and BM cells were deficient, did not develop acute liver injury. Surprisingly, mice receiving irradiation with liver shielding, in which the majority of hepatic cells were maintained similar to normal mice, developed less liver injury. These data clearly indicate that BM-derived macrophages are indispensable regardless of the existence of tissue resident macrophages.

There are three possibilities regarding the origin of CCR9^+^ inflammatory Mφs. First, CCR9^+^Mφs pre-exist in other tissues under steady state and migrate to the liver under inflammation. Second, CCR9^+^Mφs develop outside the liver under inflammation and migrate into the liver. Third, CCR9^+^Mφs originate outside the liver (i.e. circulating monocytes) and develop within the inflamed liver. Our sequential analyses in multiple tissues revealed that dramatic changes in CCR9 expression in Mφs/monocytes were only detected in the liver (Fig. 1 and Supplementary Fig. S1), suggesting that the first hypothesis is unlikely. A new concept that splenic reservoir monocytes can serve as inflammatory Mφs was recently reported in ischemic myocardial injury model^34^. However, this possibility seems unlikely in our model because CCR9^+^Mφs increased in the liver following Con A injection regardless of the existence of the spleen. Reliable surface markers that clearly distinguish hepatic-resident Mφs (Kupffer cells) from BM-derived recruited Mφs are still lacking, although enormous numbers of reported surface markers, such as CD11b, F4/80, CD68, Ly6C, and Ly6B, have been reported to date^3^,^35^,^37^. A recent comprehensive analysis to distinguish tissue-resident Mφs from recruited Mφs revealed that a combination of staining for F4/80 and CD11b can clearly distinguish these two populations in the liver both under steady state and inflammation, as CD11b^low^F4/80^high^ cells (tissue-resident) and CD11b^high^F4/80^low^ (recruited) cells^35^,^36^. We confirmed this distinction in our cells using a liver-shielded mouse model followed by BMT and subsequent follow-up for 6 weeks. As shown in Fig. 4, the hepatic CD11b^low^F4/80^high^ subset was distinct from the CD11b^high^F4/80^low^ subset reflecting the chimerism under steady state. Moreover, we demonstrated for the first time that increased CCR9^+^Mφs following Con A administration are derived from BM cells, but not hepatic-resident cells.

The molecular mechanisms of initiating inflammatory responses in the liver regarding the kinetics of infiltration by specific immune cell subset and the functional role of chemokines have been extensively investigated both in humans and different mouse models^39^. Chemokines-Chemokine receptors axis, such as CCL2-CCR2, CCL1-CCR8, and CCL25-CCR9 have been reported to promote recruitment of inflammatory monocytes/Mφs, while CX3CL1-CX3CR1 axis plays a role in limiting inflammatory functions of monocytes/Mφs^40^,^41^. However, it is still unknown how each chemokines-chemokines receptor axis contributes to the accumulation of inflammatory monocytes/Mφs. It is widely acccepted that the CCL2-CCR2 axis plays a critical role in the accumulation of BM-derived monocytes at the site of inflammation during acute liver injury^39^. Mechanistically, it was reported that Kupffer cells, hepatocytes, and activated HSCs secrete CCL2, and promote the migration of CCR2-expressing monocytes^14^. In particular, the CCL2-CCR2 axis is essential for cell egress from the BM into the peripheral circulation^42^, consistent with our data in peripheral monocytes from CCR2 deficient mice under steady state compared with WT and CCR9 deficient mice (Supplementary Fig. S3a). On the other hand, *in vivo* EdU administration data along with our previous data in hepatic Mφs from CCR9 deficient mice under inflammatory state (Nakamoto *et al*. Gastroenterology^29^ and Supplementary Fig. S3b) demonstrated that the CCR9 axis plays a novel and a specific role regarding migration and proliferation of inflammatory Mφs in the liver. Obviously, other chemokines axes play a respective role and contribute complementary during the course of acute liver injury, and further study to clarify the precise mechanism regarding this issue is required in the future.

Finally, we sought to clarify how BM-derived CD11b^+^ cells acquire CCR9 and inflammatory potential in the inflamed liver. *In vitro* experiments demonstrated that BM- and PB-derived CD11b^+^ cells, but not hepatic-resident CD11b^+^ cells, interacted with activated HSCs under inflammation and acquired CCR9 together with molecules for antigen presentation. These results are in line with our previous data that CCR9^+^Mφs interact with HSCs and promote fibrosis in the murine chronically injured liver^30^. While the role of HSCs in liver fibrosis has been well studied, their role in the pathogenesis of acute liver inflammation has not been elucidated to date. The function of HSCs was recently found to be much more diverse, as they can act as antigen-presenting cells^43^, express pattern recognition receptors^44^, respond to DAMPs and PAMPs, and have the capacity to interact with various immune cells and promote their differentiation. In this regard, it is worth mentioning that Fujita *et al*. recently showed that HSCs can mediate amplification of acute liver injury through prostaglandin signaling in both mice and humans^45^. As candidates of humoral factors from HSCs that can regulate the differentiation of BM-derived CD11b^+^ cells to CCR9^+^Mφs, we showed that retinoic acids play at least a substantial role in this process. It is well known that HSCs play an important role in the homeostasis of retinoic acids and store as much as 70% of retinols in their cytoplasm^46^. Furthermore, recent report showed that blockade of retinol metabolism by an inhibitor of alcohol dehydrogenases protected mice against Con A-induced acute liver injury^47^. Regarding the direct interaction between retinoic acids and CCR9 expression in immune cells, stimulation with retinoic acids can up-regulate CCR9 expression in T cells^48^, presumably by forming complexes with the nuclear factor of activated-T cells located at the downstream of TCR as shown recently^49^. These data support our hypothesis that BM-derived monocytes first interact with retinol-producing HSCs in sinusoids and activated CCR9^+^Mφs produce inflammatory cytokines, thereby promoting Th1 responses and subsequent acute liver injury.

Collectively, we demonstrated that inflammatory Mφs accumulating during acute liver injury originated from BM through blood circulating monocytes and became locally differentiated by interaction with HSCs. Our data provide new insights into the role of periphery derived inflammatory Mφs that is regulated by CCR9 axis. Although further comprehensive studies are still required to elucidate the molecular mechanisms underlying this interaction, our results in murine model and future verification in human samples may provide effective therapeutic potentials targeting both inflammatory Mφs as well as HSCs for acute liver injury.

## Materials and Methods

(See also Supplementary Materials and Methods for Details).

### Mice

C57BL/6 (wild-type: WT) CD45.2 mice were purchased from CLEA Japan (Tokyo, Japan). C57BL/6 CD45.1 mice and *Ccr2*^*−*/*−*^ mice were described previously^29^,^50^. All mice were maintained under specific pathogen-free conditions in the Animal Care Facility of Keio University School of Medicine. Experiments were performed with age- and sex-matched mice at 6–12 weeks of age. All experiments were approved by the animal ethics committee of Keio University, Tokyo, Japan and performed according to the guidelines.

### Con A-induced liver injury experiment

Con A (type IV) was purchased from Sigma-Aldrich (St Louis, MO). Phosphate-buffered saline (PBS) or Con A solution (20 mg/kg) was administered into the tail vein at 1, 3, 6, or 12 hours before experiments. Under anesthesia, all mice were euthanized and their serum alanine aminotransferase (ALT) levels were measured using a DRI-CHEM 3500i Analyzer (FujiFilm, Tokyo, Japan).

### Irradiation and BMT

Partial BM chimeric mice were created by shielding the liver before lethal irradiation (9.5 Gy) with 10-mm thickness of lead plate to protect hepatic-resident Mφs. Total BM cells were harvested from the femurs and tibias of age- and sex-matched WT CD45.1 mice. Isolated BM cells were suspended in RPMI 1640 medium containing 10% fetal bovine serum and 2 mM EDTA, and transplanted via the tail vein into irradiated recipient WT CD45.2 mice at a dose of 6.0 × 10^6^ cells. At 6 weeks after BM reconstruction, chimerism was confirmed by analysis of blood myeloid cells and the origin of CCR9^+^Mφs (resident vs. recruited) induced by Con A was further examined.

### Statistical analysis

Data were analyzed using JMP9 software (SAS Institute, Cary, NC) and expressed as the mean ± standard error of the mean (SEM). The Mann-Whitney *U*-test, the unpaired Student’s *t*-test and ANOVA were used as appropriate. Differences were considered statistically significant for values of *P* < 0.05.

## Additional Information

**How to cite this article**: Amiya, T. *et al*. Bone marrow-derived macrophages distinct from tissue-resident macrophages play a pivotal role in Concanavalin A-induced murine liver injury via CCR9 axis. *Sci. Rep*. **6**, 35146; doi: 10.1038/srep35146 (2016).

## Supplementary Material

Supplementary Information
