# Supplementary material for: Bone marrow-derived macrophages distinct from tissue-resident macrophages play a pivotal role in Concanavalin A-induced murine liver injury via CCR9 axis
Source: Sci Rep. 2016 Oct 11;6:35146. doi: 10.1038/srep35146 (PMC5057133; doi:10.1038/srep35146)
Supplement: Supplementary Information [file srep35146-s1.pdf]

Supplementary information

Bone marrow-derived macrophages distinct from tissue-resident macrophages play a pivotal role in Concanavalin A-induced murine liver injury via CCR9 axis

Takeru Amiya[1,2], Nobuhiro Nakamoto[1,*], Po-sung Chu[1], Toshiaki Teratani[1], Hideaki Nakajima[3], Yumi Fukuchi[4], Nobuhito Taniki[1], Akihiro Yamaguchi[1], Shunsuke Shiba[1], Rei Miyake[1], Tadashi Katayama[1], Hirotoshi Ebinuma[1], Takanori Kanai[1,*]

[1]*Division of Gastroenterology and Hepatology, Department of Internal Medicine, Keio University School of Medicine, Tokyo, Japan*

[2]*Research Unit/Frontier Therapeutic Sciences, Sohyaku. Innovative Research Division, Mitsubishi Tanabe Pharma Corporation, Yokohama, Japan*

[3]*Department of Stem Cell and ImmuneRegulation, Yokohama City University Graduate School of Medicine, Yokohama, Japan*

[4]*Department of Pathophysiology, Faculty of Pharmaceutical Sciences, Hoshi University, Tokyo, Japan*

***** Corresponding author; nobuhiro@z2.keio.jp and takagast@z2.keio.jp

Supplementary Materials and Methods

Isolation of tissue immune cells

Intestinal lamina propria mononuclear cells were separated as described previously[1]. Mesenteric lymph nodes and Peyer's patch were minced and passed through 100-μm nylon mesh. BM and peripheral blood (PB) leukocytes were hemolyzed and passed through 40-μm nylon mesh. The epididymal adipose tissue stromal vascular fraction (SVF) was prepared by digestion with 2 mg/mL collagenase type II (Sigma-Aldrich) digestion for 20 minutes. After centrifugation, the cell pellet was harvested as the SVF including adipose tissue leukocytes.

Liver and spleen mononuclear cells were isolated as described previously[2]. Briefly, livers were perfused through the portal vein and then minced and passed through 100-μm nylon mesh. The filtrate was centrifuged at 100 × *g* for 1 minute to eliminate hepatocytes with debris, and the supernatant was washed once. The cells were suspended in Hanks' balanced salt solution and overlaid on Histopaque solution (Sigma-Aldrich). After specific gravity centrifugation at 780 × *g* for 20 minutes, the cells were collected from the upper face of the Histopaque solution and subjected to flow cytometry. To determine the origin of $CCR9^{+}$Mφs, liver-resident and recruited Mφs were separated as described previously[3] with slight modifications. Briefly, livers were minced and filtered without any enzymatic digestion. The resulting cells were resuspended in 25% Percoll solution (GE Healthcare UK, Buckinghamshire, England) and gradient-centrifuged with 50% Percoll solution.

Flow cytometry analysis

After blocking with an anti-FcR antibody (CD16/32; BD Pharmingen, San Diego, CA) for 20 minutes at 4°C, cells were incubated with specific fluorescence-labeled monoclonal antibodies at 4°C for 30 minutes. The following monoclonal antibodies were used: anti-F4/80 (FITC, PE, APC), anti-CCR9 (FITC, PE), anti-MHC class II (PE), anti-CD117 (FITC), and 7-AAD (PerCP) (eBioscience, San Diego, CA); anti-Ly-6C (PE), anti-Ly-6G (FITC, PE), anti-CD11b (APC-Cy7), anti-CD11c (PE-Cy7, APC), anti-CD80 (PE), anti-CD45.1 (FITC, APC), anti-CD45.2 (APC), and lineage cocktail (including anti-Gr-1, anti-B220, anti-CD11b, anti-CD11c, anti-CD3e, and anti-CD19) (APC-Cy7) (BD Pharmingen); anti-CD115 (PE), anti-CD68 (PE-Cy7), and anti-CD169 (PE), and anti-NK1.1 (APC-Cy7), anti-CCR5 (APC), anti-CX3CR1 (PE), Siglec-H (APC), Zombie (APC-Cy7) (Biolegend, San Diego, CA); anti-CCR2 (APC) (R&D Systems, Minneapolis, MN); anti-Ly6B (PE) (Miltenyi Biotec, Bergisch-Gladbach, Germany); and anti-5-ethynyl-2’-deoxyuridine (EdU) (APC) (Molecular Probes, Eugene, OR). Each clone was summarized on Supplementary Table 1. The stained cells were analyzed using a FACS Canto II (Becton Dickinson, Rutherford, NJ), and the data were analyzed using FlowJo software (Tree Star Inc., Ashland, OR).

Splenectomy

The spleen was removed surgically under deep anesthesia. Briefly, after skin sterilization, a left-flank incision of about 5 mm was made to expose the spleen and the whole spleen was gently removed after astriction. The peritoneum and skin were separately closed with sutures. In control mice, a sham operation was performed as well as a splenectomy procedure without removing the spleen. The mice were housed for 2

weeks after surgery to allow healing and then subjected to experiments.

Hepatic resident macrophages depletion with Clodronate liposomes

In order to deplete hepatic resident macrophage, mice were injected with 200μL of Clodronate liposomes or control liposomes (FormuMax Scientific Inc., Sunnyvale, CA) 24 hours prior to Con A administration. Liver mononuclear cells extracted at 12 hours following Con A administration were analyzed by flow cytometry.

In vivo EdU uptake study

EdU labeling was performed using a Click-iT® Plus EdU Cytometry Assay Kit (Molecular Probes). Briefly, 1 mg/head EdU was intraperitoneally injected into WT mice at 10 hours after administration of Con A or PBS and allowed to incorporate into newly synthesized DNA for fluorescent labeling for 2 hours *in vivo*. At 2 hours after EdU injection, the liver and PB were collected and the cells were analyzed by flow cytometry.

Preparation of tissue extracts

The collected livers and spleens were minced, suspended in RPMI 1640 medium, and passed through 100-μm nylon mesh. The cell suspensions were sufficiently sonicated while cooling and debris was removed by centrifugation, followed by 0.22-μm polyvinylidene difluoride membrane filtration. The filtrates were collected and the total protein concentrations were measured using a BCA Protein Assay Kit (Pierce, Rockford, IL). The extracts were subdivided and stored at −80°C until use in experiments.

Isolation of HSCs and liver sinusoidal endothelial cells (LSECs)

HSCs and LSECs were separated as described previously[4]. Briefly, cells were isolated by collagenase and protease perfusion into the portal vein, followed by 8.2% and 17% Nycodenz (Accurate Chemical and Scientific Corporation, Westbury, NY) three-layer discontinuous density-gradient centrifugation. HSCs were collected from the upper face of the 8.2% Nycodenz and LSECs were harvested from the upper face of the 17% Nycodenz. The cell extracts were prepared as described above for the tissue extracts.

In vitro differentiation assay

Freshly harvested total BM cells were seeded at 5.0×10^5 cells in 96-well plates and cultured with cell extracts (7 mg/mL each) for 6 hours. The cells were then harvested, washed, and analyzed by flow cytometry.

In vitro co-culture study

After isolation of naïve BM cells (CD45.1) and HSCs from PBS or Con A treated mice liver (CD45.2), 1.7×10^6 cells of HSCs and 5×10^5 cells of total BM cells were co-cultured for 4 days in Dulbecco’s modified Eagle’s medium with 10% fetal bovine serum and 1% penicillin/streptomycin. The cells were then harvested, washed, and CD45.1 positive cells were analyzed by flow cytometry.

Fluorescence immunohistochemistry

Livers were perfused and isolated from Con A-treated mice. Part of each liver was excised, embedded in OCT Compound (Sakura Finetek, Torrance, CA) after excluding moisture, and sliced onto slide glasses (Matsunami, Osaka, Japan). The sliced sections

were fixed with acetone for 5 minutes and washed with running water. After blocking with Block Ace (DS Pharma, Tokyo, Japan) for 30 minutes, diluted anti-CCR9 (Abcam, Cambridge, UK : #ab1662), anti-F4/80 (Serotec, Oxford, UK : #MCR497R), and anti-glial fibrillary acidic protein (GFAP) (Dako, Tokyo, Japan : #IS524) antibodies were applied as primary antibodies. The sections were then incubated with appropriate secondary antibodies (Donkey Anti-Goat IgG Alexa Fluor® 488, Goat Anti-rat IgG Alexa Fluor® 568, and Goat Anti-Rabbit APC Conjugates; Invitrogen, Carlsbad, CA). After washout of excess antibodies, the sections were subjected to mounting and nuclear staining using VECTASHIELD with DAPI (Vector Laboratories, Burlingame, CA). Analysis was performed using a TCS SP5 confocal microscope (Leica, Jena, Germany).

Supplementary Table 1. Antibodies used for flow cytometry

Antigen	clone	Origin
CD11b	M1/70	BD Pharmingen
CD11c	HL3	BD Pharmingen
Ly6C	AL-21	BD Pharmingen
Ly6G	1A8	BD Pharmingen
CD80	16-10A1	BD Pharmingen
CD45.1	A20	BD Pharmingen
CD45.2	104	BD Pharmingen
Gr-1	RB6-8C5	BD Pharmingen
B220	RA3-6B2	BD Pharmingen
CD3e	145-2C11	BD Pharmingen
CD19	1D3	BD Pharmingen
F4/80	BM8	eBioscience
CCR9	eBioCW-1.2	eBioscience
MHC class II	M5/114.15.2	eBioscience
7-AAD	-	eBioscience
CD117	2B8	eBioscience
CD115	AFS98	Biolegend
CD68	FA-11	Biolegend
CD169	3D6.112	Biolegend
NK1.1	PK136	Biolegend
CCR2	HM-CCR5	Biolegend
CX3CR1	SA001F11	Biolegend
Siglec-H	551	Biolegend
Zombie	-	Biolegend
CCR2	475301	R&D systems
Ly6B	REA115	Miltenyi Biotec
EdU	-	Molecular probes

1 Hayashi, A. *et al.* A single strain of Clostridium butyricum induces intestinal IL-10-producing macrophages to suppress acute experimental colitis in mice. *Cell host & microbe* **13**, 711-722, doi:10.1016/j.chom.2013.05.013 (2013).
2 Nakamoto, N. *et al.* CCR9+ macrophages are required for acute liver inflammation in mouse models of hepatitis. *Gastroenterology* **142**, 366-376, doi:10.1053/j.gastro.2011.10.039 (2012).
3 Hettinger, J. *et al.* Origin of monocytes and macrophages in a committed progenitor. *Nature immunology* **14**, 821-830, doi:10.1038/ni.2638 (2013).
4 Chu, P. S. *et al.* C-C motif chemokine receptor 9 positive macrophages activate hepatic stellate cells and promote liver fibrosis in mice. *Hepatology* **58**, 337-350, doi:10.1002/hep.26351 (2013).

Supplementary Figure legends

Supplementary Figure S1 CCR9^{+}Mφs are not pre-existing in steady state. Representative CD11b and CCR9 staining on total isolated mononuclear cells of liver, spleen, BM, colon, SI, PB, WAT, MLN, and PP from mice at indicated time point pre-and post-Con A administration.

Supplementary Figure S2 CCR9^{+}Mφs are originated from BM via blood circulating monocytes without contribution of hepatic resident Mφs. (a) Sequential change in the absolute number of PB mononuclear cells from control (n=2), shield + BMT (n=3), and TBI + BMT (n=3) mice up to 6 weeks. (b) Frequency of CD68, CD169 (markers for Kupffer cells) and CCR2, Ly6B (markers for recruited Mφs) on CD11b^{low}F4/80^{high} resident Mφs and CD11b^{high}F4/80^{low} recruited Mφs. Data show mean ± SEM (n=5). (c) Irradiated mice with a whole liver shield (shield) were reconstituted with BM cells. 1 weeks after BM transplantation, mice were further injected with Con A. Correlation between the chimerism of PB monocytes and the chimerism of CCR9^{+} Mφs (white squares) or CCR9^{-} Mφs (black squares) 12 hours after Con A injection (n=4 each). (d) Representative CCR9 and CD11b staining on whole mononuclear cells in the liver (left) and the frequency of CCR9^{+}CD11b^{+} macrophages at 12 hours following Con A administration in control and clodronate pre-treated mice(right) (n=4-6) ** :p<0.01.

Supplementary Figure S3 Migration of monocytes/ Mφs is differently regulated by CCR2 and CCR9 axis. (a) Left: Representative CD11b and Ly6C staining on PB leukocytes in WT, *Ccr2*$^{-/-}$, and *Ccr9*$^{-/-}$ mice. Right: Percentage of Ly6C^{++}CD11b^{+} monocytes in total PB leukocytes. Data Show mean ± SEM (n=4) (b) Left: Representative CD11b and CD11c staining on hepatic mononuclear cells in WT, *Ccr2*$^{-/-}$, and *Ccr9*$^{-/-}$ mice. Right: Percentage of CD11b^{+} Mφs in hepatic mononuclear cells. Data Show mean ± SEM (n=4). * : p<0.05, ** : p<0.01. n.s. : not significant.

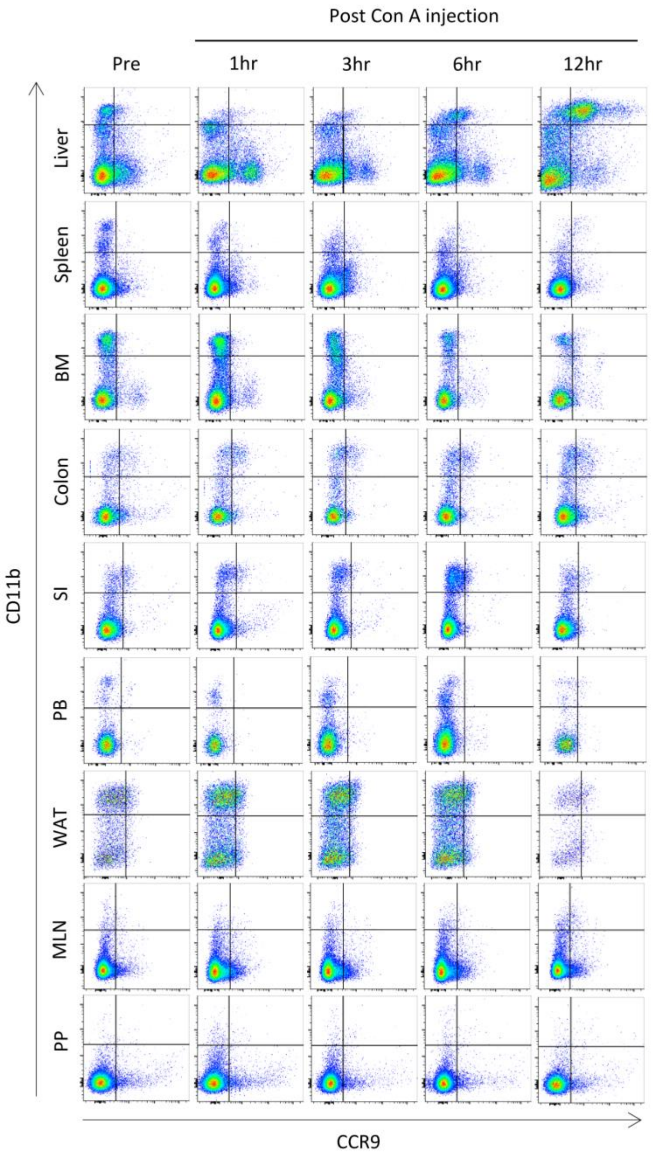

Supplementary Figure S1

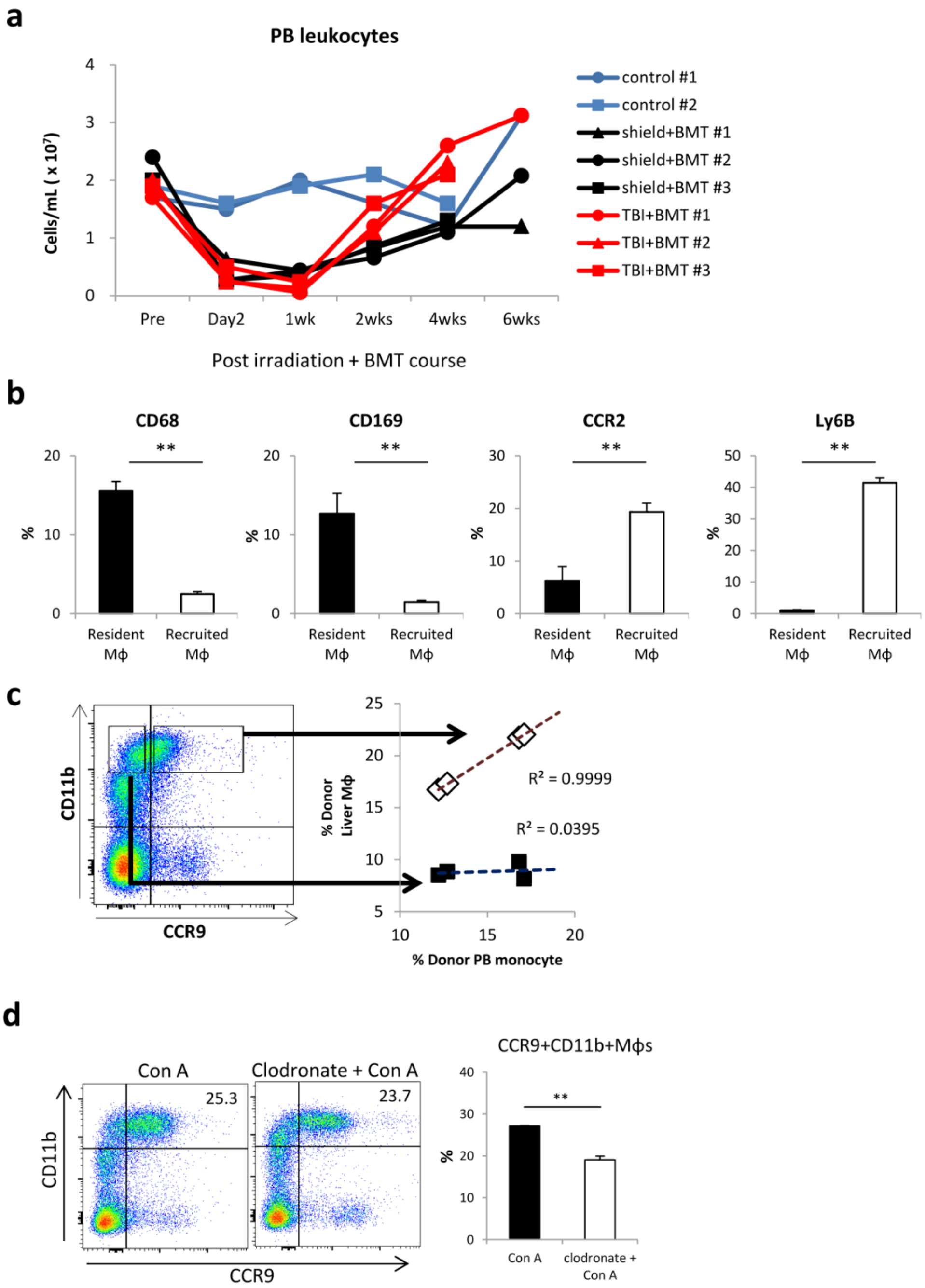

Supplementary Figure S2

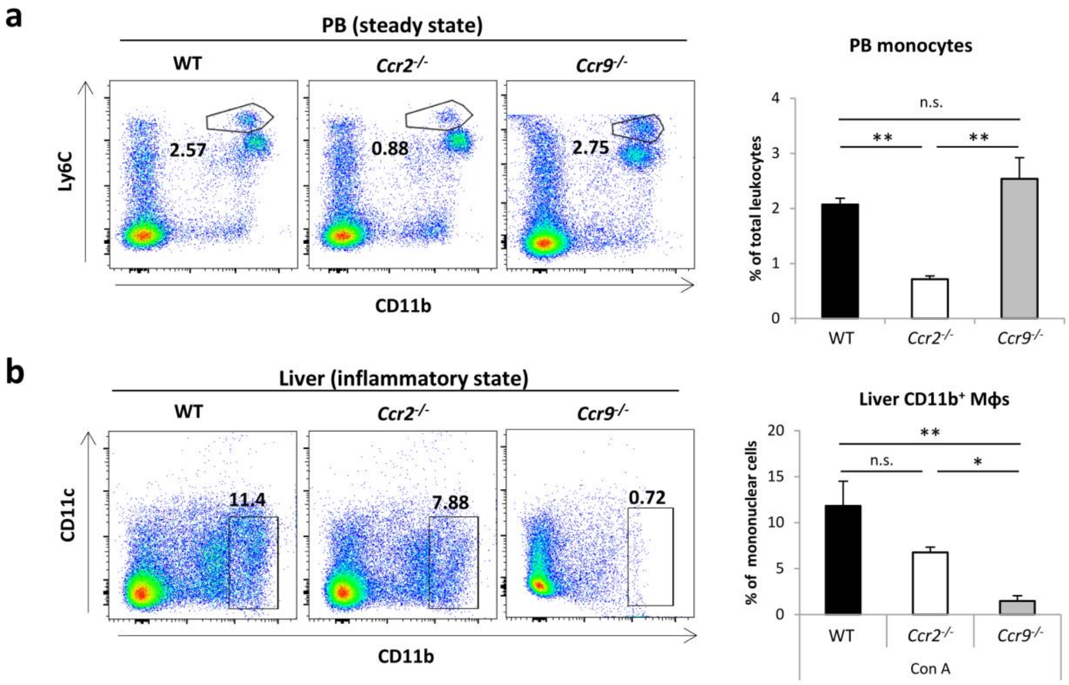

Supplementary Figure S3
